# Electroactive Polymers Obtained by Conventional and Non-Conventional Technologies

**DOI:** 10.3390/polym13162713

**Published:** 2021-08-13

**Authors:** Akel F. Kanaan, Ana C. Pinho, Ana P. Piedade

**Affiliations:** CEMMPRE, Department of Mechanical Engineering, University of Coimbra, 3030-788 Coimbra, Portugal; akel.kanaan@dem.uc.pt (A.F.K.); acdspinho@uc.pt (A.C.P.)

**Keywords:** electro-responsive polymers, conventional fabrication, additive manufacturing, polymer processing and performance, electroactive material, 4D printing

## Abstract

Electroactive polymers (EAPs), materials that present size/shape alteration in response to an electrical stimulus, are currently being explored regarding advanced smart devices, namely robotics, valves, soft actuators, artificial muscles, and electromechanical sensors. They are generally prepared through conventional techniques (e.g., solvent casting and free-radical polymerization). However, non-conventional processes such as those included in additive manufacturing (AM) are emerging as a novel approach to tune and enhance the electromechanical properties of EAPs to expand the scope of areas for this class of electro-responsive material. This review aims to summarize the published work (from the last five years) in developing EAPs either by conventional or non-conventional polymer processing approaches. The technology behind each processing technique is discussed as well as the main mechanism behind the electromechanical response. The most common polymer-based materials used in the design of current EAPs are reviewed. Therefore, the main conclusions and future trends regarding EAPs obtained by conventional and non-conventional technologies are also given.

## 1. Introduction

Electroactive polymers (EAPs) are electro-responsive materials capable of converting electrical energy/information into physical response/perturbation (e.g., bend, swell, shrink). EAPs can change their size or shape when subjected to an applied electrical stimulus. They belong to the class of “smart” materials such as the pH-, light-, temperature-, enzymatic-, ultrasound-, redox-, ionic strength- and magneto-responsive materials.

EAPs have recently attracted significant scientific and technological interest due to their potential to be applied in the design of advanced electronic systems, namely soft actuators, artificial muscles, electromechanical sensors, robotics, and micro-valves/pumps [1,2,3,4].

In response to applied electrical stimuli, electro-responsive polymer-based materials generally undergo polymer collapse, electrochemical reactions, ionic-polymer-metal interactions, or changes in electrophoretic mobility [5]. That response is intimately dependent according to several variables, including temperature, amount of electro-/ion-responsive entity source, nature of the polymeric network, the polymer’s degree of crosslinking, the shape and thickness of the polymeric electro-responsive material, pH and ionic strength of surrounding media, intensity of the applied electrical stimulus, presence of plasticizers, and experimental setup (position of the electrodes relative to the electro-responsive material). Furthermore, the type of processing of electro-responsive materials also plays an essential role in determining the final properties and performance of the final material [6]. Most current EAPs systems have been designed by conventional processing technologies, namely solvent casting and free-radical polymerization. The utilization of non-conventional procedures such as additive manufacturing (AM) techniques in the design of EAPs is a less explored area and can be employed to fabricate complex and novel electro-responsive systems with defined architecture and shape, and improved performance with a high degree of reproducibility. However, AM technologies are more restricted in terms of the variety of polymers that can be used to develop EAPs when compared to those available for conventional ones.

This review focuses on the processing and performance description of recent polymer-based electroactive systems obtained by conventional and non-conventional processing techniques to highlight the new directions/trends and limitations in engineering advanced electro-responsive polymer-based systems for several applications. A brief description of the mechanisms behind relevant applications of electroactive systems is presented. Moreover, an overview (from the last five years) of the most commonly employed conventional and non-conventional processing techniques is given in the following sections and the description of several EAP systems organized by their constituent polymer.

## 2. Mechanisms behind Relevant Applications of EAP Materials

### 2.1. Actuator

EAPs are lightweight and flexible materials (low Young’s modulus) that can perform an electro-assisted mechanical displacement (bending) to different extents according to the applied electrical stimulus. This class of electro-responsive materials can be divided into two groups: electronic EAP and ionic EAP [7].

**Figure 1:**
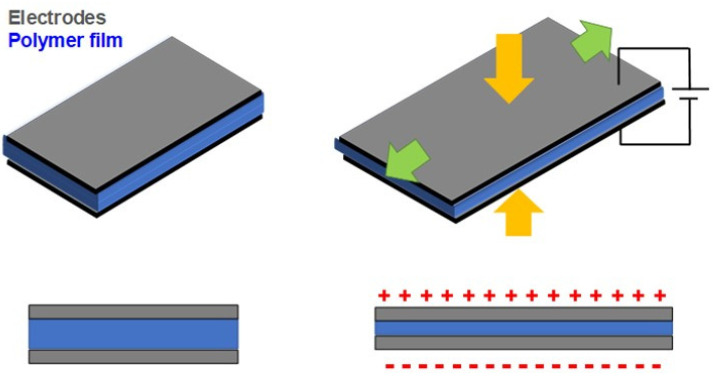
Schematic illustration of electromechanical actuation of a nonionic EAP dielectric elastomer.

Electronic EAPs, also referred to as non-ionic EAPs, are polymer-based actuators that can work under dry conditions and requires high electrical voltages (>1 kV) to operate. Additionally, they are often constituted from an insulating elastomer membrane (dielectric elastomer, DE) (e.g., PVC, poly(urethane) (PU), and natural rubber) between flexible/stretchable electrodes (e.g., carbon or silver grease) deposited on both its sides. Its bending mechanism is described by coulombic attraction (Figure 1). The application of high electrical stimulus generates an attractive force between two electrodes, squeezing the polymer membrane in the thickness direction leading to the collapse of the elastomer that ultimately results in mechanical displacement. This effect is reversed when the electrical stimulus is ceased.

**Figure 2:**
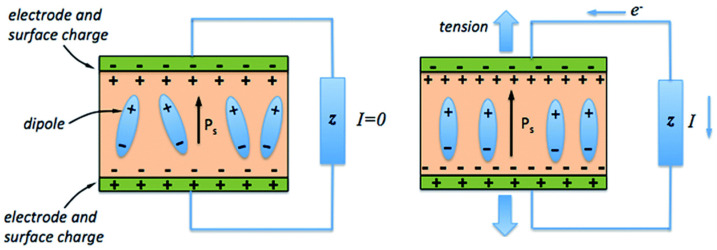
Electro actuation mechanism of a piezoelectric polymer actuator [11]. Open access, Copyright Royal Society of Chemistry.

Another example of electronic EAPs is piezoelectric actuators. Piezoelectric polymers can convert an electrical signal into mechanical actuation (converse piezoelectric effect) or mechanical stimuli (compression, vibration) into electrical energy (direct piezoelectric effect). For a given polarized piezoelectric polymer, its electro-assisted mechanical displacement is given by the ordered rearrangement of the electric dipoles along the poling direction (Figure 2) [8]. This electro-induced dipole reorientation within the polymeric matrix induces polymer lengthening or contraction according to the polarization of the applied electrical stimulus and, thus, electromechanical actuation. Poly(vinylidene fluoride) (PVDF) is the most studied polymer in developing lightweight actuators [9,10].

Ionic EAPs actuators are polymer actuators that present electro-induced mechanical displacement due to ion diffusion/migration within the polymeric matrix. They present bending motion under lower applied voltages (< 5 V) and generally presented higher bending displacement (electromechanical actuation) when compared to electronic EAPs [7]. Ionic EAPs are commonly composed of a polymeric network (neutral or a polyelectrolyte matrix) loaded/soaked with electrolytes (including ionic liquids (ILs)) and/or carbon nanotubes (CNTs). Examples of this class of actuator include: conducting polymer actuators [9,12], ionic-polymer metal composite (IPMC) actuators [13,14] and CNT actuators [15,16].

Although the mechanisms of electro-responsive actuation of ionic EAPs present some similarities, each specific case (depending on the chemical nature/composition of the material) requires its proper description.

**Figure 3:**
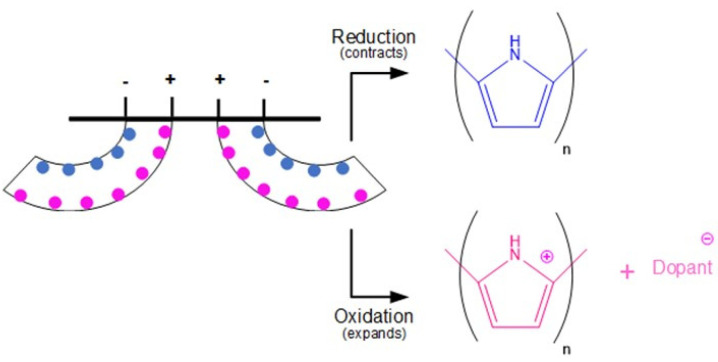
Schematic illustration of the anion-driven electro actuation mechanism of PPy.

Conjugated polymer-based electro actuators (e.g., poly(pyrrole) (PPy), poly(aniline) (PANI), poly(acetylene) (PAc) and poly(thiophene) (PTh) and its derivatives (such as poly(3,4-ethylene dioxythiophene) and polystyrene sulfonate, PEDOT:PSS) have their actuation response driven by complex electrochemical redox reactions at the polymer/electrode interface upon electrical stimulation (Figure 3). Briefly, considering polymer PPy doped with a generic anion (Dopant-), reduction of PPy takes place at the cathode (negatively charged electrode), reducing PPy to its neutral state, expelling dopant ions resulting in polymer contraction. Conversely, oxidation reactions at the anode (positively charged electrode) side protonate PPy where dopant ions are inserted (to compensate for charge imbalance), resulting in polymer expansion. This electro-induced volume change results in electromechanical displacement towards the cathode (also referred to as anion-driven actuation).

Cation-driven actuation can occur in specific cases such as PPy/dodecylbenzene sulfonate (DBS) systems following a similar mechanism explained earlier for an anion-drive. However, expansion/contraction is given at the cathode/anode resulting in electromechanical displacement towards the anode. Bending actuation performance can be tuned as a function of several variables, including applied polarization, temperature, the intensity of the electrical stimulus, type and size of the dopant, among others [17].

In the case of polyelectrolyte-based EAPs, such as IPMCs, electro-assisted mechanical displacement is governed by four main mechanisms, namely coulomb [18], electroosmosis [19], electrochemical [20], or dynamic enrichment/depletion mechanisms [21].

**Figure 4:**
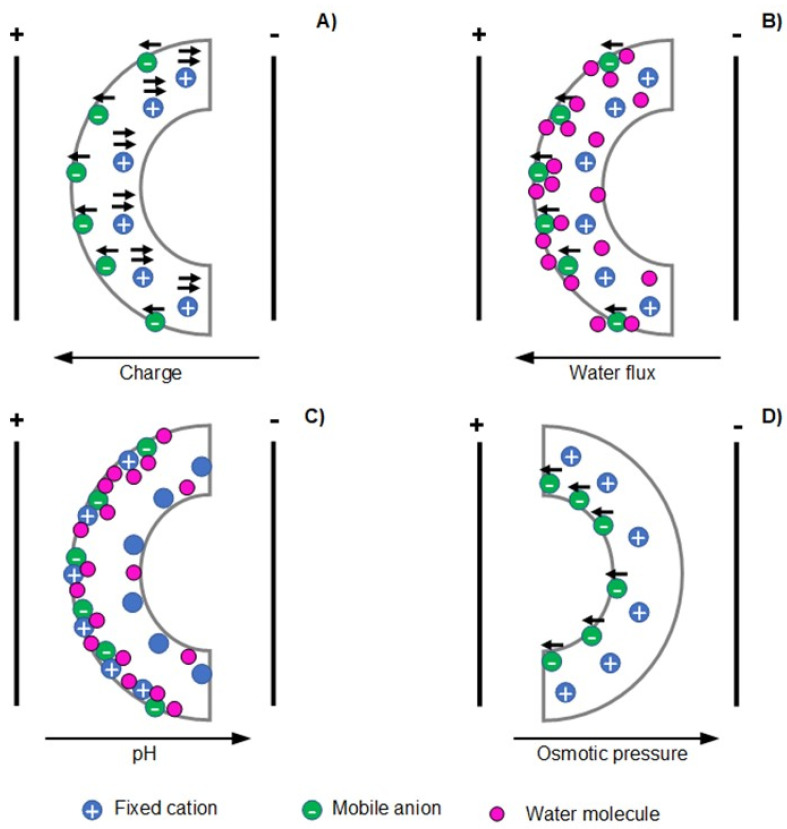
Representation of electro-assisted mechanical displacement mechanisms for a polycationic actuator. Coulomb (**A**), electroosmosis (**B**), electrochemical (**C**) and enrichment/depletion (**D**) mechanism.

Figure 4 illustrates the aforementioned bending mechanisms taking as an example a polycation [22,23]. The coulomb mechanism describes the electro-assisted actuation by the net force exerted (by the electrical stimulus) on fixed and mobile ions of the polyelectrolyte matrix, causing a stationary current inside the polymer that ultimately pulls the polymer towards the cathode (Figure 4A).

Electroosmosis mechanism describes the electro-driven actuation response by the electrophoretic diffusion of hydrated counter ions (from the polyelectrolyte network) that leads to local swelling (expansion) at the side-facing anode and local shrinking (contraction) at the side-facing cathode electrode (Figure 4B). The electrochemical mechanism is related to the differences in pH (induced by the electrolysis reactions of water) in the vicinity of the electrodes. These pH changes alter the redox states of the polyelectrolyte actuator, which affects its swelling equilibrium resulting in volume changes that leads to bending motion towards the cathode (Figure 4C). The gradient in osmotic pressure causes the enrichment/depletion mechanism of actuation at the solution/polymer interface induced by the dynamical accumulation or depletion of ions at this interface. As a result, the osmotic pressure gradient affects the swelling/shrinking capacity of the polyelectrolyte at its surface vicinity, leading to bending towards the anode (Figure 4D).

**Figure 5:**
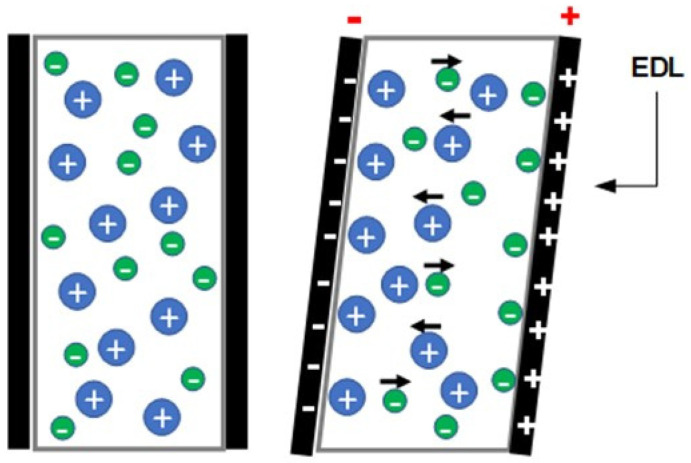
Illustration of EDL bending mechanism.

The electrical double layer (EDL) formation mechanism is often utilized to describe the electro-assisted actuation profile of CNTs-based actuators, ionogel, and electro-double layer actuators. Briefly, by the appliance of electrical stimulus ions (both cations and anions), electrophoretic diffuse towards oppositely charged electrode accumulating at the polymer/electrode interface resulting in the formation of an EDL (Figure 5). When cations are larger than anions, polymer expansion is attained at the cathode side, while polymer contraction is obtained at the anode side. These local volume changes induced by the size difference between ions generate uniaxial stress along with the polymer matrix resulting in bending motion towards the anode [24,25,26].

### 2.2. Sensor

The electroactive capacity of EAPs has also been explored in the development of elec-tromechanical sensors (e. g. haptic/tactile devices and blood pressure/pulse rate monitor-ing) [4]. Moreover, EAPs are also referred to in the literature as promisor materials for elec-trochemical sensing [1]. However, since the sensing mechanism behind this type of sensor is commonly related to specific factors (such as electronic/ionic conductivity, cou-pling/binding of a biological/synthetic analyte resulting in a physicochemical change) in-stated of conductivity variation induced by mechanical stimulus (resulting in size/shape alteration), they are not considered in the present paper.

In general, the electromechanical sensing mechanism of electronic and ionic EAPs is related to its electro-assisted mechanical displacement mechanism (as previously described). For these systems, a correlation between mechanical perturbation (stress) and electrical conductivity is envisaged.

**Figure 6:**
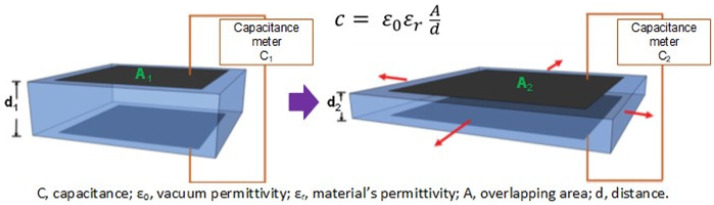
Illustration of electromechanical sensing mechanism for DE [4]. Open access, Copyright Royal Society Publishing.

In electronic EAPs such as DE and piezoelectric actuators, the electromechanical sensing mechanism is given: DEs present their electromechanical sensing capacity by measuring the capacitance across the elastomer after the mechanical deformation (compression and stretching) [4]. As the magnitude of the measured capacitance is in the function of area and thickness of the elastomer when the DE has mechanically deformed, an alteration in capacitance created by the electrodes is obtained (higher capacitance for larger areas and lower thicknesses) (Figure 6).

Piezoelectric electromechanical sensors present their sensing mechanism according to the previously mentioned direct piezoelectric effect. Moreover, the electromechanical sensing mechanism of these materials is intimately related to the chemical structure/configuration of its polymeric network. For instance, a polarized PVDF sensor containing an all-trans configuration between fluorine and hydrogen atoms (β-phase) is given. An external mechanical input application decreases the magnitude of polarization due to the distance reduction between PVDF’s fluorine and hydrogen atoms. Consequently, when the mechanical input ceases, the voltage output is obtained due to the increased distance between fluorine and hydrogen atoms [4]. Finally, this electrical output is recorded by a voltage amplifier, and its magnitude correlates with the polymer’s form alteration/deformation by the mechanical stimulus.

**Figure 7:**
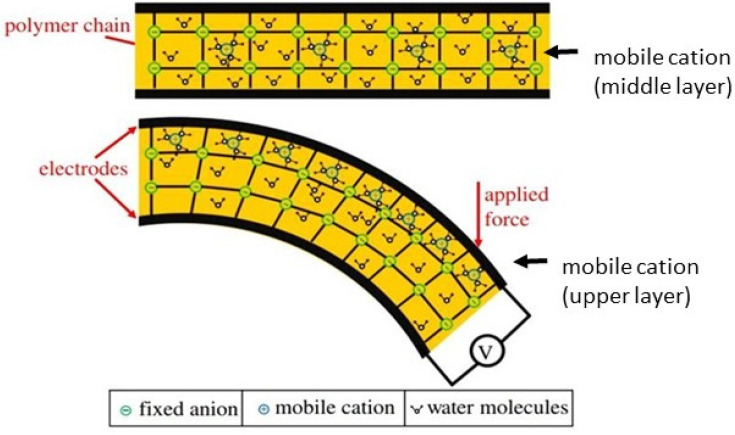
Schematic representation of electromechanical sensing mechanism of a typical IPMC [4]. Open access, Copyright Royal Society Publishing.

In general, ionic EAPs present a similar electromechanical sensing mechanism based on the uneven distribution of mobile ions (within the polymer network) upon mechanical deformation. For instance, for a classical IPMC, upon mechanical stimulus (such as bending and stretching), mobile ions diffuse from the contract/stretched region to the expanded area (in order to counterbalance charge distribution) (Figure 7). As a result, this distribution of ions originates a charge gradient along with the polymer’s bulk, inducing a potential difference proportional to the induced mechanical perturbation [27]. Finally, this voltage output is recorded by a low-power amplifier, for example.

### 2.3. Drug Release

EAP materials were investigated in developing different drug-delivery systems. Several mechanisms are related to electro-assisted drug delivery, namely electrophoresis/electroosmosis mechanism [28] and forced convention mechanism [29]. Since the former mechanism is not related to electro-induced size and shape alteration of the polymeric drug reservoir, it is not considered in the present review. Conversely, the forced convention mechanism is described by releasing the drug from the polymeric network due to deswelling/shrinkage (in the case of a pH-responsive polyelectrolyte) or bending/polymer constriction (in the case of an actuator) along with water syneresis.

**Figure 8:**
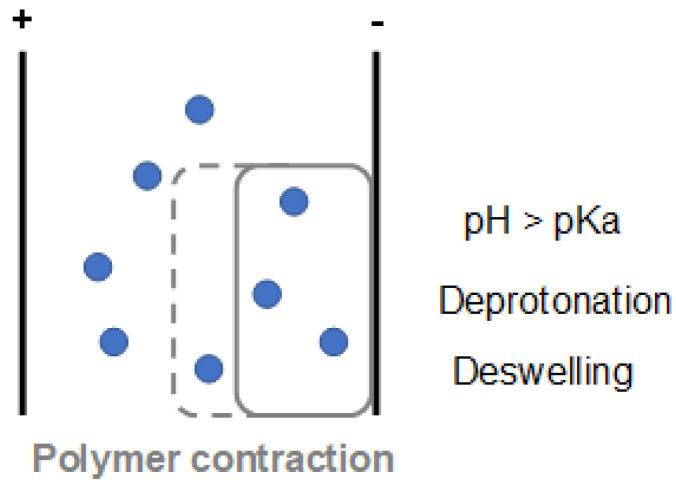
Electro-assisted drug delivery by forced convention mechanism for a polycationic material.

For example, a drug-loaded polycation mounted on the cathode gives an electro-assisted forced convention mechanism: when the electrical stimulus has applied, the pH at the electrode’s vicinity increases (due to the water electrolysis reactions), inducing the deprotonation of alkaline groups of the polycationic material. This deprotonation leads to the reduction of the interchain free-volume (by lowering the electrostatic repulsion among polymer’s chains) and, consequently, polymeric matrix deswelling/shrinkage (Figure 8). This mechanical deformation induces the expulsion of the drug content from the polymeric material by squeezing it out by the forced convection mechanism [29]. Finally, drug delivery from an electro-actuator polymeric material follows the same trend after that electromechanical displacement (bending). The constricted area squeezes out the drug by the aforementioned mechanism [30].

## 3. Conventional Technologies and Most Common Polymers to Produce EAPs

Several conventional technologies were employed to develop EAPs envisaging different applications, namely spin coating, complexation, photopolymerization, copolymerization, electropolymerization, solvent casting, and free radical polymerization. Nevertheless, the solvent casting and free radical polymerization are the most utilized conventional polymer processing techniques in the design of EAPs due to their simplicity, low cost, versatility, and high efficiency.

### 3.1. Solvent Casting

**Figure 9:**
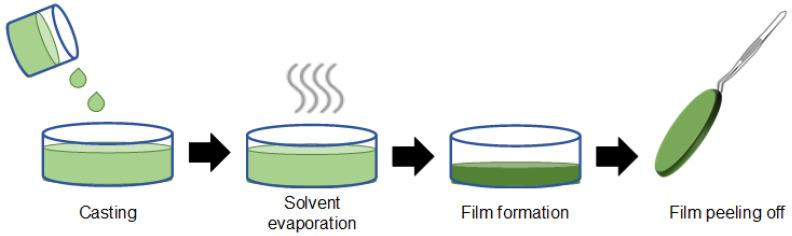
Illustration of film formation by conventional solvent casting.

Solvent casting is one the most straightforward polymer processing techniques widely applied in the design of EAPs. Figure 9 shows polymer-based thin film being obtained by a conventional solvent casting procedure. Firstly, a given polymer is dissolved in a volatile solvent such as water or organic solvent (often dimethyl sulfoxide (DMSO), dimethylformamide (DMF)) to obtain a homogeneous viscous solution. That solution is poured on a mold (casting) and conducted in an oven to evaporate the solvent (solvent evaporation). Consequently, a thin polymer-based film is obtained after the drying process (film formation) and peeled off the mold [31,32]. Moreover, the format and thickness of the material obtained can be adjusted according to the shape of the mold utilized and the polymer solution’s volume. Finally, the properties of the final material can be tuned by the presence of plasticizers, soluble drugs, additives in the casting step by the type of solvent utilized in the polymer solution that can render materials with different viscosities and porosities [33].

The disadvantage of this procedure is that the obtained materials are often restricted to applications in the open air. Polymer network dissolution and/or additive leaching occur in aqueous environments since no chemical reactions (e.g., crosslinking or grafting) are present.

### 3.2. Free-Radical Polymerization

Conventional free-radical polymerization (non-controlled polymerization) is the most frequently applied polymerization technique employed in the development of EAPs due to its ease of implementation, high tolerance towards several functional organic groups, and impurities in the reaction solution.

**Figure 10:**
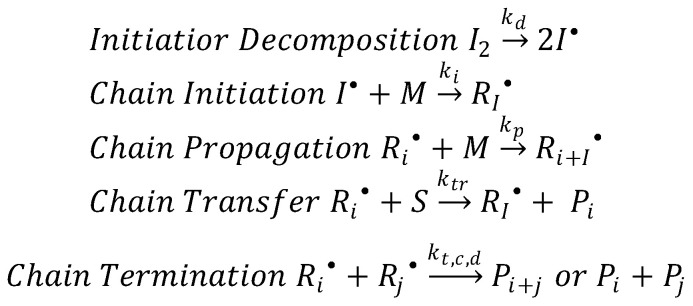
Schematic representation of conventional free-radical polymerization mechanism, where *I*_2_ is the initiator, *k* is the kinetics, *M* is the monomer, *R_i_*^•^ is the chain length’s radical, *S* is a transfer agent, and *P* is the polymer. *P_i+j_* and *P_i_* + *P_j_* refer to termination by combination and disproportionation, respectively.

This technique involves the polymerization of monomers in solution, commenced by generating free radicals (from a free radical source), which further produces propagating radicals, to obtain random polymeric networks. Generally, free-radical polymerization can be described by the following chain mechanisms (as shown in Figure 10): (1) initiation (generation of primary radicals); (2) propagation (radical addition from initiators to monomers yielding primary propagating radicals); (3) chain transfer (increase in the number of polymer molecules and decrease in the degree of polymerization) and (4) termination by the combination (radical-radical combinations) or disproportionation (atom abstraction reactions between active chains) [34].

Briefly, free radicals are generated by thermal or photochemical homolytic cleavage of covalent bonds or by a redox process. These generated free radicals are added to (“attack”) vinylic bonds of the monomer (initiation), resulting in the formation of primary propagating radicals that further rapidly propagates (propagation) in order to form radical centers. In the presence of a transfer agent (S), the radical centers are transferred from the polymer end to another molecule yielding a dead polymer and a small radical (chain transfer). Finally, when the propagating radicals come into relative proximity of one another, deactivation of these propagating radicals takes place either by combination or disproportionation (termination) [34,35].

The most commonly used initiators are 2.2′-azobisisobutyronitrile (AIBN), potassium persulfate (KPS), dibenzoyl peroxide (BPO), and 2-hydroxy-4′-(2-hydroxyethoxy)-2-methylpropiophenone (Irgacure 2959). The type and amount of employed initiator play a significant role in determining the molecular weight of the final material due to its influence on the rate of polymerization and structure [34]. Finally, the conventional free-radical polymerization mechanism is behind several polymerization process: homopolymerization, copolymerization, in situ polymerization, crosslinking, and grafting.

### 3.3. Polymers Used in EAPs

#### 3.3.1. Poly(Vinylidene Fluoride) (PVDF)

PVDF and its copolymers are the most studied polymer utilized in the development of EAPs due to their low price, versatility, high polarity, easy processing, piezoelectric responsiveness, and dielectric constant. It is constituted as long polymeric chains between fluorine and hydrogen atoms (covalently bonded by carbon atoms) that commonly exists in three distinct polymorph phases (α, β, and γ) being the β-phase (all-trans configuration) the one that presents higher electroactive-responsiveness [36]. Additionally, PVDF can be combined with different compounds such as ILs, CNTs, and graphene oxide (GO) in order to obtain electroactive materials envisaging different applications, namely actuators [9], haptic sensors [37], and drug-delivery systems [30].

Correa et al. studied IL’s cation head size’s influence on the bending performance of electroactive PVDF-based actuators [9]. PVDF/IL composites were prepared by solvent casting in DMF with several ionic liquids from different families, such as imidazolium, piperidinium, pyrrolidinium, and ammonium (at a fixed amount of 40wt%), sharing the same counter ion (bis-(trifluoromethylsulfonyl)imide, (TFSI)).

**Figure 11:**
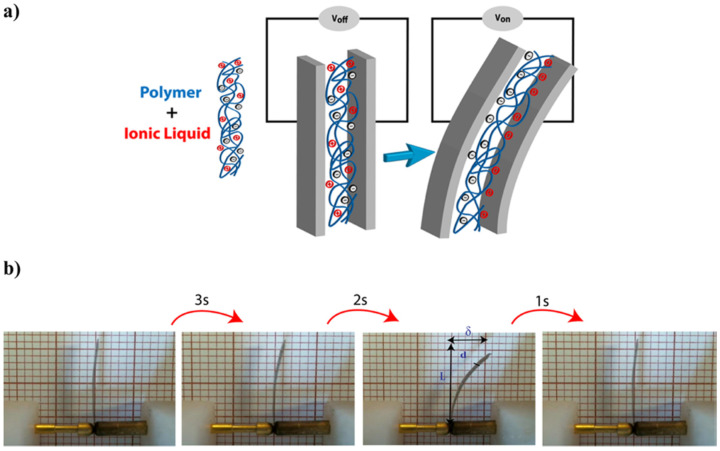
Electromechanical displacement illustration due to ion migration (**a**), and bending motion profile as a function of time for PVDF/[PMIm][TFSI] composite at 5V and 100 mHz (**b**) [9]. Reprinted with permission of the American Chemical Society.

Their results demonstrated that electromechanical displacement was greatly influenced by the cation head size of the ionic liquid, where higher bending motion was observed for PVDF/IL composites containing 1-methyl-1-propylpiperidinium [PMPip]+ and 1-propyl-3-methylimidazolium [PMIm]+ cation heads at 5 V and 100 mHz. Its bending mechanism is due to the ion motion within the PVDF matrix in response to applied electrical stimulus (Figure 11a), as previously described for a typical EDL actuator.

The actuation profile also demonstrated a time-dependency (Figure 11b) behavior where the polymer actuator regained its original position/shape within a short period when the electrical stimulus was ceased. Moreover, PVDF/IL composites loaded with higher molecular weight ILs (greater alkyl chain length of the cation head) presented lower Young’s modulus (due to IL’s plasticizing effect), which resulted in a more flexible polymeric network that favored bending by lowering the mechanical resistance of the material. Finally, electromechanical displacement is often non-symmetric between cycles (when compared to the initial position of the actuator) for this type of electro-actuators due to charge imbalances (related to the size differences between cations and anions) that lead to different ion mobilities/diffusivities within the polymer network when negative/positive electrical inputs are applied [38].

Recently, PVDF-based piezoelectric films were developed regarding biomedical applications as drug-delivery systems. Zhang and co-workers prepared PVDF-co-hexafluoropropylene (PVDF-HFP) films with reduced graphene oxide (rGO) via solvent casting in DMF. The resulting films were further chemically modified at the surface by a combination of plasma treatment and layer-by-layer technique in order to obtain coated poly(allylamine hydrochloride)/polyamidoamine (PAH/PAMAM) crosslinked multilayers PVDF-HFP films [30].

**Figure 12:**
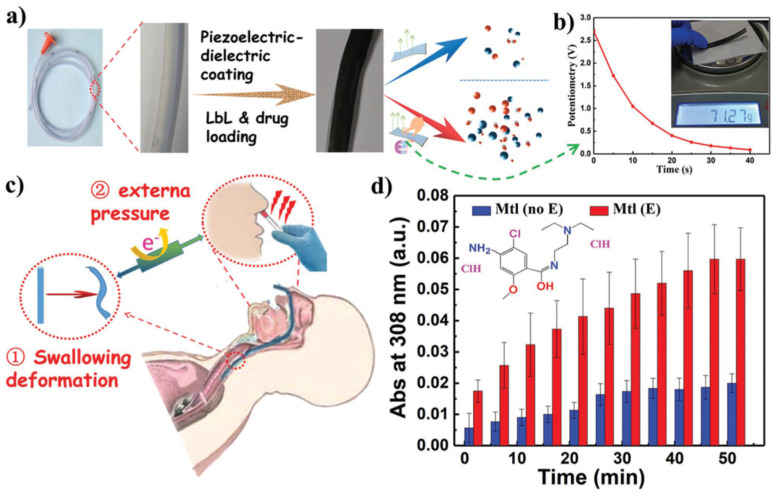
Illustration of the obtained PVDF-HFP based films (**a**), time-dependent piezoelectric potential curve (**b**), schematic representation of piezoelectric mechanism response upon application (**c**), and metoclopramide release profiles obtained with and without mechanical stimulation (**d**) [30]. Reprinted with permission of the Royal Society of Chemistry.

They studied the release profile of metoclopramide (an anti-nausea/vomiting cationic drug) in phosphate-buffered saline (PBS) upon external mechanical stimulus towards medical applications, namely gastric lavage process. The idea was to take advantage of the mechanical deformation of the gastric tube during the intubation process in the patient’s stomach to enhance local drug delivery. Figure 12a shows in detail the PVDF-HFP based films loaded with metoclopramide. Upon mechanical stimulus (pressure of 0.7 N that mimics the pressure exerted by human swallowing), the material could generate time-dependent small electric potential outputs of 2.7 V (Figure 12b).

This piezoelectric response (induced by mechanical deformation of the polymer matrix as seen in Figure 12c) enhanced the release profile of the cationic drug by 200% for 60 min compared to those obtained without the mechanical stimulus (Figure 12d). The mechanism of metoclopramide release from the PVDF-HFP based films can be associated with cationic drug electrophoretic diffusion, in response to internally generated voltage upon external pressure, and with the forced convection mechanism as previously explained.

#### 3.3.2. Nafion^TM^

Sulfonated tetrafluoroethylene-based fluoropolymer-copolymer, also known as NafionTM, is an anionic polyelectrolyte that is often employed in developing ionic EAP actuators due to its ionic conductivity, chemical, and mechanical stability, and ease of processability. Electro actuators of NafionTM/1-ethyl-3-methylimidazolium tetrafluoroborate (NafionTM/EMImBF4) composites were prepared in N-dimethylacetamide (DMAc) by the solvent casting technique [39]. Consequently, layers of single-walled carbon nanotubes (SWCNTs) were added to each surface side of the resulting composites by hot-pressing to obtain a sandwich structure.

The electro actuating response of prepared Nafion^TM^-based materials was investigated under different variables, including DC voltages (1–4 V), waveforms (square, sinusoidal, triangular, and sawtooth), and frequencies amplitudes (0.01–100 Hz) in the open air. Firstly, Nafion^TM^-based composites presented reversible bending motion towards the anode upon electrical stimulation due to size differences between EMImBF4 ions following the EDL mechanism presented earlier. Results demonstrated that a linear relationship between the intensity of the applied electrical stimulus and bending displacement amplitude. As expected, the increase in voltage input led to higher/faster ion mobility within the polymeric matrix resulting in a higher bending motion. Similarly, the effect of waveforms onto bending motion followed the trend where higher signal excitations (square waveform) resulted in higher electromechanical displacement when compared to lower signal energy (sawtooth waveform) at ±4 V. Conversely, an inverse relationship was found between bending motion and frequency amplitude due to shortening of applied voltage (±4 V) response time as the frequency augments [39].

Another crucial parameter that needs to be considered when developing EAP actuators is the electromechanical displacement profile between cycles to evaluate the given actuator’s mechanical stability (durability) upon prolonged repetitive stimulation. This parameter is not often evaluated [13,14]. However, it should be considered in further studies. The same work previously described [39] also studied the life cycle of NafionTM-based actuator in response to the electrical stimulus of ±4 V square wave at 1 Hz in the open air. Results demonstrated that the electro actuator could withstand at least 20,000 cycles during ~5 h of experiments presenting a 20% reduction in strain (compared to initial strain at t = 0 h) which could be related to the annealing of the constituent materials of the given actuator.

#### 3.3.3. Poly(2-acrylamido-2-methyl-1-propanesulfonic acid) (PAMPS)

PAMPS is an anionic polyelectrolyte commonly utilized in the design of ionic EAPs due to its low price, hydrophilicity, and ion conductivity. Moreover, through simple polymer processing techniques such as free-radical polymerization, 2-acrylamide-2-methyl-1-propanesulfonic acid) (AMPS) the monomer can be homopolymerized/copolymerized/crosslinked with different compounds to obtain complex polyanionic structures. Li et al. developed tough and stretchable PAMPS-based hydrogels for applications as soft actuators [40]. Hydrogels were prepared by free-radical copolymerization of aqueous solutions of acrylamide (AAm) with AMPS crosslinked by Pluronic^®^ diacrylate (F127DA) in glass molds at 60 °C for 24 h. The electromechanical displacement capacity of the obtained hydrogels was evaluated in saline aqueous media at different salt concentrations and intensities of applied electrical bias. As expected, a linear relationship between the intensity of the applied electrical stimulus and bending displacement response was observed at a fixed salt concentration [0.05 M]. The increase of applied electrical voltage led to an augment in mobile ion diffusion rate (inside the hydrogel and in the aqueous media), which resulted in higher/faster electromechanical motion. The polyanionic hydrogels presented a reversible bending angle towards the cathode (equivalently given by each polarity) following the enrichment/depletion mechanism as previously explained.

The effect of electrolyte concentration in ionic EAPs that operates in aqueous media is an essential factor for the design of soft actuators regarding more complex environments such as in vivo. There is no direct relationship between the increase in mobile ions concentration in the solution (inside the hydrogel, at equilibrium) and electromechanical displacement response. Low concentrations of ions in aqueous media led to a significant decrease in the content of mobile ions, which results in lower bending angle rates. Conversely, if the concentration of ions is too high, the diffusion of mobiles ions induced by the electrical stimulus is hampered (due to the shielding effect), leading to lower response rates and, ultimately, lower bending angles. The highest bending angles (87°) were observed for sodium sulfate (Na_2_SO_4_) concentration of [0.05 M] (at a fixed applied voltage of 20 V) while at [0.01 M] and [0.15 M] bending angles were 59° and 30°, respectively [40] due to mechanism previously described. More recently, Ying et al. also demonstrated this trend for novel electroactive PAMPS-based double network hydrogels [41]. Their results demonstrated that the electromechanical displacement response was faster for intermediate values of ionic strengths when compared to those observed for 0.01 and 0.15 M.

#### 3.3.4. Poly(pyrrole) (PPy)

PPy is an electro-conductive polymer widely explored in the design of ionic EAP actuators for several applications, namely soft robotics, artificial muscles, and conductive scaffolds for tissue engineering [42,43]. Despite its high conductivity, PPy has some drawbacks, including low mechanical properties (e.g., brittleness and lack of flexibility) and low water handling capacity (e.g., high hydrophobicity and low solubility), which are pivotal depending on the envisaged application. Nevertheless, these disadvantages are often overcome by conjugating PPy with more flexible and/or hydrophilic polymers such as chitosan, poly(ethylene glycol) (PEG)/poly(ethylene oxide) (PEO), gelatin, among others.

PPy-based materials can be obtained by conventional free-radical polymerization or by electropolymerization. Since the latter is specifically employed in the polymer processing of this class of material, it was not covered in Section 3 of this review, where it represents the majority-utilized polymer processing techniques in general. However, a brief explanation is given: electropolymerization relies on the formation of monomer radicals induced by the electro-assisted redox reactions at the electrodes. Low voltages (100–200 mV) are applied through the monomer solution generating monomer radicals that further combine resulting in a progressive polymer film deposition at the surface of the working electrode [44]. The advantage of this technique is that it avoids the utilization of extra catalysts and chemical initiators to trigger/induce the polymerization process.

PPy-based electromechanical actuators were recently developed regarding the design of soft actuators for robotics. Khadka et al. synthesized PPy-PEO actuators by electropolymerization technique and studied the role of PEO content (5–20wt%) on its bending response. Electromechanical displacements were performed in bis(trifluoromethane) sulfonamide lithium (LiTFSI) aqueous solutions under consecutive potential cycles (200 cycles) in the range of −0.6 – +0.6 V at a scan rate of 5 mV/s [42]. Their results demonstrated that electromechanical displacement behavior was inversely proportional to the PEO content in PPy-based actuators, where higher PEO content decreased (~1.3x lower) in bending motion due to the decrease in charge density and conductivity as the PEO content increases. The bending motion was towards the anode, presenting an expansion at the cathode (reduction) and contraction at the anode (oxidation) typical for a cation-driven actuation response.

**Figure 13:**
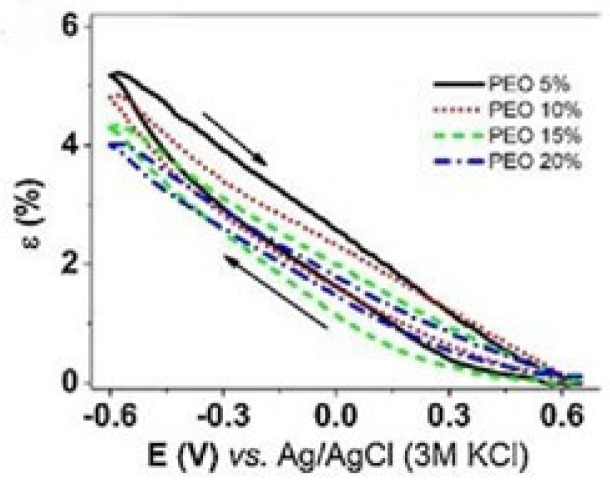
Strain (ε) profile of PPy/DBS-PEO materials in LiTFSI aqueous media with different contents of PEO and of CFS-PPyTF in CCM electrolyte [42]. Reprinted with permission from Elsevier.

Moreover, cyclic actuating voltammetry experiments revealed stable responses (closed cycles). However, asymmetric bending (measured by strain ε (%)) was observed between cathodic/anodic scans for the same applied voltage (Figure 13). This behavior is due to differences in electrically triggered redox-based conformational and structural changes (shrinking/swelling) and also to solvent exchange asymmetries (influx of ions from the aqueous media by osmosis coupled with electrophoretic migration of ions from the polymer gel to the solution) [42].

Another interesting work was developed by Harjo et al. for the design of conductive fiber scaffolds (CFS) that can perform electromechanical actuation in a complex environment such as cell culture medium (CCM) regarding tissue engineering applications [43]. Glucose-gelatin nanofiber scaffolds (obtained by electrospinning) were further coated with PPy (doped with trifluoromethanesulfonate CF3SO3-, CFS-PPyTF) by electropolymerization. Cyclic voltammetry actuation experiments were conducted in CCM within the voltage range from −0.6 to + 0.6 V. Their results demonstrated that CFS-PPyTF materials could perform electromechanical displacement in CCM, presenting a cation-driven bending motion. Furthermore, the stability and durability of PPy-based actuators were evaluated under square wave potential steps at a fixed frequency of 0.1 Hz (600 cycles) in the same voltage range previously described in CCM. After 600 cycles (~1.7 h), a reduction in strain was observed (~25% lower) when compared to the initial strain at t = 0 h [43].

Interventional studies involving animals or humans, and other studies that require ethical approval, must list the authority that provided approval and the corresponding ethical approval code.

More studies need to be performed to fully address the factors that influence the electromechanical actuation performance in complex environments, such as CCM.

#### 3.3.5. Poly(aniline) (PANI)

PANI is another electroconductive polymer employed mainly in developing ionic EAPs as conductive electrodes due to its low price, ease of synthesis, and high conductivity. PANI-based materials are generally designed to envisage applications as soft actuators, electronic devices, and artificial muscles [45,46].

Sun et al. developed PANI-based nanocomposite soft actuators with high electromechanical performance in media’s pH and amount of PANI [45]. Soft actuators were prepared by depositing PANI/multi-walled carbon nanotubes (MWCNT)/reduced graphene oxide (rGO) composite solution (at various pH values) via spray coating (naturally dried at room temperature) onto the surface of cellulose/IL electrolyte membranes. Electromechanical displacement experiments were conducted at a voltage bias of 5 V in the open air. Upon electrical stimulus, the nanocomposite rapidly bent towards the anode due to the ion diffusion (cations and anions from the IL) within the polymeric matrix, following the mechanism mentioned above for ionogels. Compared to the blank (material with no PANI content), the presence of PANI greatly enhanced the electromechanical displacement of tested membranes presenting a displacement of ~5 cm for both directions according to the polarization applied.

Nevertheless, the further increase of PANI content led to a decrease in bending response due to the augmenting membrane’s hardness (higher mechanical resistance) which influenced its flexibility and compromised the electromechanical displacement. Moreover, the influence of pH also played an important role in determining the bending profile. More acidic environments (pH of 2.2–2.5) led to a higher electromechanical displacement when compared to higher pH values of 3.6 due to a higher ion conductivity (lower charge transfer resistance) which promoted higher ion flow within the polymeric network resulting in higher bending motion [45].

In another interesting work, Beregoi et al. developed PANI-based microtubes as building blocks for artificial muscles applications [46]. The electropolymerization of aniline monomers prepared the microtubes (though periodic voltage pulses of 0 V and 1 V during 120 s) onto the surface of gold (Au) coated electrospun poly(methyl methacrylate) (PMMA) fibers. Resulting PANI-coated PMMA/Au fibers had their PMMA content removed by immersion in dichloromethane to obtain hollow PANI/Au microtubes. Bending measurements were performed in simulated gastric fluid (SGF) by applying polarized electrical stimulus between −0.2 and 1.0 V during 5 cycles. Upon electrical bias, the microtubes presented asymmetric bending towards the cathode following the anion-driven mechanism previously explained. This asymmetry could be associated with a stress gradient in the polymeric structure that induced a more significant electromechanical displacement in a particular direction.

Additionally, the authors also evaluated the mechanical stability and durability of PANI/Au actuators under electromechanical stimulation during 200 potential cycles at the same voltage range previously mentioned. Their results demonstrated that developed actuators were stable; however, they presented a slight decrease after ~100 s of measurements, probably due to the over-oxidation of PANI (threshold of 0.6 V). Finally, the physical integrity of PANI/Au microtubes was evaluated after 200 potential cycles by scanning electron microscopy (SEM) micrographs where no surface cracking was observed in the Au layer, and PANI film became more rugged [46].

#### 3.3.6. Poly(3,4-ethylenedioxythiophene) Poly(styrene sulfonate) (PEDOT:PSS)

Poly(thiophene) derivative, PEDOT is a largely explored electron-conducting polymer in the development of electronic devices such as actuators [47] and sensors [48] due to its high conductivity, easy processability, and thermal stability.

Recently, PEDOT:PSS-based ionic EAPs with high performance was developed by Lu et al. regarding applications such as wearable electronics and soft actuators [47]. Flexible electrodes were prepared as follows: PEDOT:PSS-based films were prepared by solvent casting (at 60 °C under vacuum) comprising aqueous solutions of PEDOT:PSS, ethanol (5%), ethylene glycol (5%), and surfactant 607. Therefore, the obtained films were deposited on each surface side of an IL-loaded poly(urethane) (PU) membrane by hot pressing resulting in an EAP sandwich structure. Electromechanical displacement measurements were conducted under varying sine wave electrical stimulus of ±0.1–±3.0 V at 0.1 Hz in the open air. Results demonstrated that bending motion was oriented towards the anode due to the diffusion of IL’s cations and anions inside the polymeric network following the actuation mechanism of ionogels as previously described. The electromechanical displacement behavior presented a linear relationship with the magnitude of the applied electrical stimulus where higher bending motion was observed for higher voltage inputs. As expected, higher electrical stimulus (±3 V) induces faster ion diffusion/motion within the polymeric matrix resulting in a faster/greater electromechanical response (displacement of ~10 mm). Conversely, with the increase in frequency from 0.1 to 5 Hz (at fixed voltage sine wave bias of 3 V), lower electromechanical displacements (up to 4xsmaller) were observed due to the reduction of ion migration response time [47].

Zhang et al. developed transparent, stretchable PEDOT:PSS-based pressure-responsive systems for potential electromechanical sensor applications as electronic skin [48]. The patterned spin-coating deposition (at 500 rpm and 1500 rpm, for 30 s each) was used with PEDOT:PSS Clevios PH1000 aqueous mixture, glycerol (5% v/v), and surfactant Capstone FS-30 (1%) onto the surface of poly(dimethylsiloxane) (PDMS) substrates through a poly(ethylene terephthalate) (PET) mask. Films were subsequently dried at 100 °C (during 1 h) for solvent evaporation. The materials obtained presented different PEDOT:PSS-based film thickness (of 130 and 50 nm) according to the amount of solution deposited onto PDMS sheets. Electromechanical sensing experiments were conducted by monitoring the electrical conductivity (under a constant applied voltage of 1 V) of developed films at different applied strains (15–60%) as a function of PEDOT:PSS-based layer thickness. Their results demonstrated a linear relationship between the film thickness and electromechanical sensing capability. As the thickness of the PEDOT:PSS layer decreases, lower current variation between the stretched and the released state was observed, resulting in smaller electromechanical sensing capacity. Interestingly, in thicker films (130 nm), large surface cracks upon stretching were observed during measurements. These physical defects hampered the current passage through the film (tortuosity effects), which allowed a fine recording of current variation and, thus, distinct mechanical sensing (in order of magnitude of 10 Pa, which resembles gentle finger touch) [48].

#### 3.3.7. Poly(vinyl chloride) (PVC)

PVC is another classical polymer utilized mainly in developing nonionic EAPs. Its low cost, ease of processing, and versatility allow its conjugation with different compounds/additives, resulting in a final material with distinct electrical and mechanical properties. Recently, PVC-based durable electro-actuator gels with high-performance were developed for promising applications as artificial muscles and soft actuators [15,49]. Shin et al. developed robust and transparent PVC-based electro actuator gel with bending motion behavior dependent on PVC’s molecular weight [49]. The electro-responsive materials were prepared by solvent casting comprising a solution of PVC (at different molecular weights: low (116.000), medium (239.000) and high (282.000), plasticizer dibutyl adipate (DBA) in tetrahydrofuran (THF). The homogeneous mixture obtained was poured into Teflon^®^ dishes, dried at room temperature for 72 h, and vacuum dried for 24 h. In the open air, the resulting transparent films were evaluated in electromechanical displacement capacity under high electrical potentials (~1 kV). Results showed that bending motion was towards the anode, and it followed a similar mechanism previously presented for piezoelectric actuators. By applying an electrical stimulus, dipoles of DBA reorganize and become oriented according to the polarization inducing a poling force towards the PVC polymeric chains, resulting in bending motion. The gels presented an “on-off” behavior during bending measurements recovering the original position when the electrical stimulus was ceased.

Moreover, electromechanical experiments demonstrated that the bending motion of PVC gels was dependent on its molecular weight, and greater (~3.6x higher) electromechanical displacements were observed for high and medium molecular weight (HMW and MMW, respectively) when compared to the PVC of low molecular weight (LMW). This behavior was attributed to the higher density of the HMW PVC polymeric network that prompted more significant interaction with DBA’s dipoles under electrical stimulation resulting in higher bending motion displacements. Finally, cyclic electromechanical performance experiments revealed that PVC gel actuators were durable after 25,000 cycles (~3 days of stimulation) where no significant difference in maximal bending displacement (180°) was observed [49].

Ali et al. developed highly electro-responsive PVC-based actuators comprising DBA-plasticized PVC and functional multiwall carbon nanotubes (FMWCNTs) [15]. Soft composites were prepared by solvent casting with several amounts of incorporated FWCNTs. Briefly, PVC:DBA (1:4 *w*/*w*%) was dissolved in THF, and then FMCNTs (compositions of 0.01, 0.02, and 0.03% relative to the total mass) were added. The resulting solution was poured into Petri dishes and left for 3 days for solvent evaporation, where soft composites were obtained. Electro-induced mechanical deformation of the materials containing different FMCNTs content was carried under an applied voltage of 1 kV in the open air. Electromechanical displacement experiments showed that response time and bending angle were significantly improved for PVC gels with higher FMWCNTs content. The higher amount of FMWCNTs prompted faster and higher electrical charge transfer through the PVC gels, which induced rapidly reorientation of DBA dipoles (following the mechanism previously mentioned) that ultimately resulted in greater electromechanical displacements (up to ~1.8x greater) and shorter response times (up to ~3.5x lower) [15].

#### 3.3.8. IL-Based Polymers/Poly(ionic liquids) (PILs)

PILs have been explored in the development of advanced EAPs for soft electromechanical-responsive platforms. They belong to a unique sub-class of polyelectrolytes with permanently charged groups, independent of the media’s pH (due to the absence of acidic/alkaline groups as in aprotic ILs), improved mechanical properties, high durability, and processability when compared to pure ILs. PILs/IL-based polymers conjugate the properties of the ionic liquids (e.g., ionic conductivity, negligible vapor pressure, and thermal stability) with those from polymers (e.g., functionalization, viscoelastic properties, and 3D structure) [50]. Recently, IL-based copolymers ionic EAPs were developed for soft actuators and promising tissue engineering scaffolds applications [12,51].

Kokubo et al. developed IL-based copolymers ionic polymer actuators and studied the influence of polyelectrolyte structure (polycationic or polyanionic) in the bending performance [51]. Crosslinked polymer electrolytes were prepared by free-radical copolymerization of cationic IL monomer (1-[(3-methacryloyloxy)propyl]-3-methyl imidazolium bis(trifluoromethanesulfonyl)amide, [PMIm-MA][TFSA]) or anionic IL monomer (1-methyl-3-propylimidazolium N-[3-(methacryloyloxy)propylsulfonyl]-N-(trifluoromethanesulfonyl)amide, [PMIm][TFSA-MA]) with poly(ethylene glycol) methyl ether methacrylate (PEGMEN) plasticizer, crosslinked by ethylene glycol dimethacrylate (EGDMA, 2 mol% of the total monomers). Then, initiator 2,2′-azobis(isobutyronitrile) (AIBN) was added, and the solution obtained was poured into PET molds separated by Teflon^®^ spacers. The reaction was carried at 80 °C for 24 h and at 120 °C for 4 h to ensure complete polymerization. The resulting crosslinked IL-based copolymers were sandwiched between flexible carbon electrodes by hot pressing at 170 °C for 10 min. Bending displacements were recorded by the applied electrical bias of a ±2 V rectangular wave with a fixed frequency of 0.01 Hz in the open air. Electromechanical displacement measurements revealed that bending actuation direction and displacement depended on the fixed charge type of the IL-based polyelectrolyte. The bending direction was governed by the counter-ion motion of materials where polycations and polyanions bent towards cathode and anode, respectively, following the electroosmosis mechanism previously mentioned. Moreover, polyanions presented higher (~1.3x greater) bending displacement than polycations due to differences in the size of counter ions of each polyelectrolyte. Polyanion’s counter ions (PMIm+) were smaller (~1.2x smaller) than polycation’s (TFSA-), which facilitated its diffusion through the polymeric network in response to an electrical stimulus resulting in higher ionic conductivity and also, greater electromechanical displacements [51].

**Figure 14:**
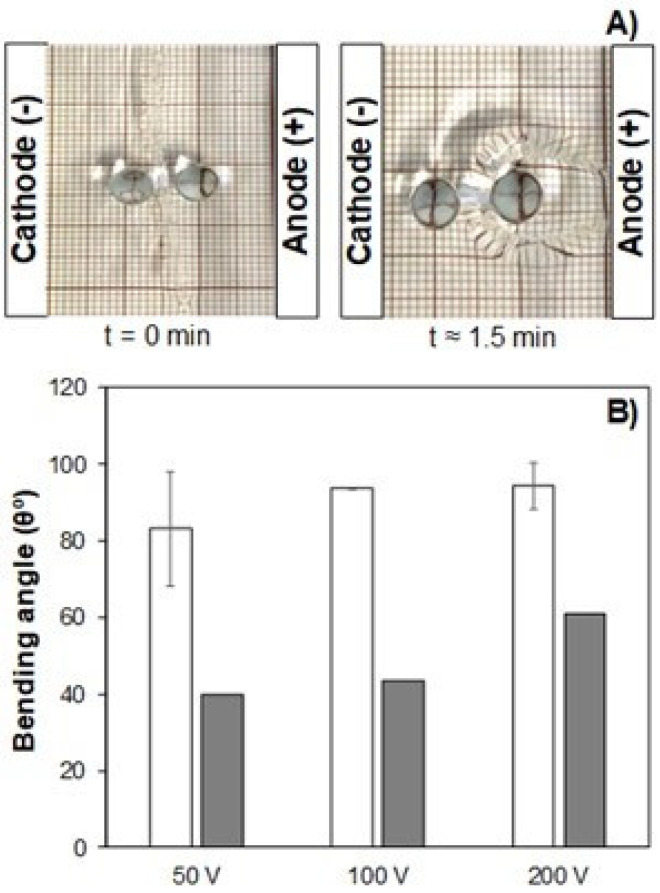
Illustration of T-T hydrogel actuation response under 100 V (**A**), and bending angles at the different applied electrical stimulus for T-T (white columns) and T-G (grey columns) IL-based polycationic hydrogels (**B**). Experiments were conducted in deionized water at room temperature (~22 °C) [12]. Reproduced with permission from Elsevier.

Kanaan et al. evaluated mold assemblies-induced interfaces in the electromechanical actuation performance in the developed IL-based polycationic hydrogels [12]. Crosslinked soft actuators reinforced with silica nanoparticles were prepared by free-radical copolymerization of 2-hydroxyethyl methacrylate (HEMA, 9.27 mmol) with 1-butyl-3-vinylimidazolium chloride (BVImCl, 3.10 mmol) in an aqueous solution containing the crosslinker N,N’-methylenebis(acrylamide) (MBA, 0.5 mol% of total monomers) and MCM-41 silica nanoparticles (0.4 *w*/*w*% of total monomers). Then, the initiator ammonium persulfate (APS, 0.2 molar% of total monomer) was added. The homogeneous solutions obtained were poured into different mold assemblies (separated by 1 mm silicon spacers) with different hydrophilicity, such as Teflon^®^ and glass substrates. Free-radical polymerization was carried in an oven at 50 °C (for 8 h) and then at 70 °C (for 24 h) to ensure complete polymerization. The resulting hydrogels were washed in deionized water (to remove unreacted monomers) and further coded after the type of substrate utilized during the polymerization reaction, namely Teflon^®^-Teflon^®^ (T-T), glass-glass (G-G), and Teflon^®^-glass (T-G). Bending displacement measurements were performed at different voltage biases (of ±50, 100, and 200 V) in several aqueous media such as deionized water, acidic solution (pH = 5.0), and alkaline solution (pH = 9.0). Electromechanical actuation results demonstrated that bending performance was highly influenced according to the type of mold utilized during polymerization. T-T hydrogels presented greater (at least ~ 1.5x greater) bending angles than T-G hydrogels with bending orientation towards the anode (according to the enrichment/depletion mechanism previously mentioned) (Figure 14A,B).

Conversely, G-G hydrogels presented no electromechanical displacement (at a macroscopic level) for all tested voltage ranges. The results indicated that electromechanical displacement depended on the surface-to-bulk effect induced by the mold substrate used during polyelectrolyte synthesis. Less-crosslinked/loser surfaces were observed for T-T hydrogels, while denser/compact structures were obtained for G-G. Therefore, T-G hydrogels presented a combination of the aforementioned physical characteristics. T-T hydrogels’ softer and the less-crosslinked surface allowed a faster counter ion diffusion (in response to applied electrical bias), resulting in higher bending displacements (equivalently given for each polarization). In the case of T-G hydrogels, Cl- counter ions diffusion (from the IL) was hampered when the glass side of the hydrogel was facing the cathode due to the higher density of the surface that was in contact with glass during polymerization that ultimately resulted in lower bending angles. The durability of T-T hydrogels was evaluated for at least 24 h where its actuating response was consistently maintained during experiments. Finally, electromechanical actuation performance was not significantly influenced by the media’s pH (comparing the results obtained for deionized water with acidic/alkaline aqueous media) since IL-based polyelectrolyte hydrogels do not present ionizable groups along with its polymeric network [12].

#### 3.3.9. Cellulose

Cellulose, the most abundant natural polymer in nature, has recently been employed to develop ionic EAPs actuators [52] and sensors [53]. This polysaccharide received significant attention in the design of wearable electroactive devices due to its biocompatibility, low cost, hydrophilicity, adhesiveness, and chemical versatility (allow the conjugation/functionalization with several compounds/additives to render different physicochemical and electrochemical properties).

In the work of Teresawa and co-workers, the authors could develop flexible and transparent cellulose-based PEDOT:PSS/IL gel actuators with high performance [52]. The actuators were developed by solvent casting technique composed of a sandwich structure of piperidine-functionalized cellulose nanofibers (PCN) dissolved in imidazolium-based IL complied between two PCN/PEDOT:PSS/IL flexible electrodes. Electromechanical displacements were carried at triangular waveforms of ±2 V in the open air. Bending experiments demonstrated that different strains under applied electrical stimulus could be obtained according to the chemical nature of the ionic liquid utilized and PEDOT:PSS content which affected the gel’s ionic conductivity and mechanical properties. Moreover, electro actuating behavior followed the EDL mechanism previously described. Finally, the gels presented high durability and mechanical stability under cyclic experiments (at least 10,000 cycles) at ±1 V and 1 Hz [52].

Self-powered electromechanical sensors capable of detecting human motion (finger bending, exhalation, and speech) were developed by Wang et al. [53]. Ion conducting cellulose-based hydrogels were obtained by in situ free-radical polymerization. Briefly, cationic nanocellulose (CNC) was complexed with anionic graphitic carbon nitrile (g-C3N4) in NaCl solution. The complex obtained was added to acrylamide (AAm) solution containing MBA, APS, and tetramethyl ethylenediamine (TMEDA) as a crosslinker, initiator, and catalyst, respectively. The resulting CNC-based poly(acrylamide) (PAAm) gel was poured into molds followed by solvent evaporation in the oven at 60 °C for 12 h. Strain sensor testing measured the resistance upon mechanical loading (strain) in a copper wire-hydrogel-linked system connected to an electrochemical station. Results demonstrated that CNC-based hydrogels presented sensibility to a variety of mechanical stimuli, namely compression (loads of 75 and 200 g), strain (200%), finger bending (bent and relaxed position), and thought movement (by loud and low voice tones). The electromechanical sensibility arises from the ion transport imbalances (from the g-C3N4-CNC complex) following the aforementioned mechanism during mechanical stimulation. For example, due to shape change upon stretching, a local decrease in ion concentration takes place (imbalances in ion diffusion/rearrangement within the hydrogel), which results in higher resistivity when compared to its initial state (without mechanical solicitation), allowing different and distinguishable current variation profiles to be created [53].

#### 3.3.10. Chitosan (CS)

Chitosan, a chitin derivative, is the second most abundant biopolymer after cellulose and also is the unique naturally occurring polycationic polysaccharide. Moreover, CS has been employed in developing ionic EAPs as polymer actuators and artificial muscles [16,54] due to its easy processability, low cost, ionic conductivity, biocompatibility, and non-toxicity.

Sun et al. developed electroactive and biocompatible chitosan-based electro actuators presenting fast response and performance [54]. Chitosan-based actuators loaded with 1-butyl-3-methylimidazolium tetrafluoroborate (BMImBF4) IL electrolyte sandwiched by MWCNT layers were prepared by solvent casting. Briefly, homogenous solutions (15 mL) of CS [2%] dissolved in acetic acid with BMImBF4 and MWCNT were obtained and further processed by solvent casting (at 60 °C) to produce thin (40–50 μm) MWCNT electrodes. CS-based ionogel was prepared by solvent casting by dissolving 2 mL of IL and 1 mL of glycerol into acidic CS [2%] solution (20 mL). The films were obtained by solvent casting (at 80 °C) and further complied between previously prepared MWCTN-based electrodes by hot pressing resulting in a 200–250 μm thick sandwiched structure. Electromechanical displacements were carried at a constant applied DC voltage of 5 V as a function of different MWCNT contents (20–80%). Results demonstrated that CS-based actuators presented bending towards the anode following the EDL mechanism previously presented. A linear relationship was observed during electro-actuation experiments between the MWCNT content in the electrode layers and the bending magnitude. Higher amounts of MWCNT (80%) resulted in higher conductivities which induced higher and faster ion diffusion (from IL and protonated chitosan counter ions) within the polymeric matrix that ultimately resulted in higher (up to ~7x greater) electromechanical displacement with faster (up to ~3x faster) response time [54].

Bio-inspired CS-based artificial muscles were developed by Zhao et al. [16]. Crosslinked CS-based actuators sandwiched by flexible MWCNT electrodes were prepared by solvent casting. In short, MWCNT electrodes consisted of homogenous solutions (20 mL) of CS [0.6%] dissolved in acetic acid with 5 mL MWCNT aqueous dispersion and were poured into glass molds and subject to solvent casting under vacuum at 80 °C (for 6 h). Crosslinked CS-based hydrogels were obtained by dissolving 2 mL of glycerol in acidic CS [3%] solutions (20 mL) containing different amounts of the crosslinker genipin. These solutions were poured above the previously prepared dried MWCNT electrode layers at 60 °C for 8 h. Therefore, another layer of MWCNT electrode solution was evenly deposited on the surface of crosslinked CS-based hydrogel, where sandwiched films were obtained by solvent casting. Electromechanical experiments were conducted at an applied DC voltage of 5 V in the open air, and the effect of crosslinking density on bending performance was evaluated. Results demonstrated that bending motion was oriented towards cathode due to the diffusion of mobile protonated CS’ counter ions (CH_3_COO-) following the electroosmosis mechanism previously explained. Overall, compared to the electromechanical results obtained for non-crosslinked samples, CS-based actuators with the highest crosslinking density presented lower (at least ~1.2x smaller) mechanical displacements and slower (up to ~2x slower) response speed. Finally, these results indicate that the increase in crosslinking degree augments Young’s modulus (lower mechanical flexibility), hindering the free movement of ions within the polymeric network resulting in lower electromechanical displacements with slower response performances [16].

## 4. Non-Conventional Technologies and Most Common Polymers to Produce EAPs

Materials science is a field of research at constant evolution. Proof of that is the advent of a series of processes included in additive manufacturing (AM) that revolutionize the concept of building parts. Briefly, AM techniques allow producing highly complex parts, layer by layer with a bottom-up approach, from previously drawn CAD/CAM objects [55]. After the digital design is ready, a slicer software “slices” the object into layers with controllable height creating a new file known as gcode, then it is read by the AM equipment chosen.

Besides shape accuracy, surface finishing features, and reliability, the advent of AM represents enormous progress as regards ambiental issues. Indeed, it is considered green technology due to the lack of waste production [56]. Furthermore, the different AM techniques do not require a high power supply or specific room conditions.

For all these reasons, the study and employment of AM have been increasing exponentially in recent years in a wide range of application fields. AM has been associated with 4D printing in the present manuscript, which refers to objects able to change their shape upon an external stimulus.

The study of materials processed by AM that can respond to an electrical stimulus is still in an early stage, which explains the lack of work published compared to conventional technologies described earlier. Nonetheless, as mentioned, it is an unexplored field with a high progress margin. The following sections present a brief overview of the currently reported AM techniques used in processing the EAPs and provide some examples of materials and approaches already published.

### 4.1. Fused Filament Fabrication (FFF)

**Figure 15:**
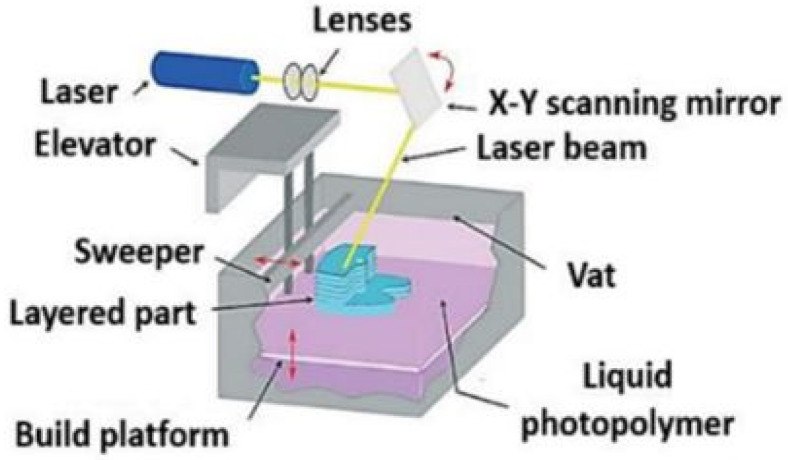
Three-dimensional (3D) printer apparatus scheme [59].

Fused filament fabrication (FFF) is a technique based on the extrusion of materials that allows the production of intricate parts within short periods using apparatus known as 3D printers [57]. For this reason, from all AM techniques, FFF is probably the most cited and recognized among the science community and general society. The working principle is relatively easy to understand and does not require specialized tooling or handling, facilitating its spreading among users. In addition, after patent expiration, 3D printers have been developed by many manufacturers with very competitive prices accessible to the public [58,59,60,61,62]. A schematic representation of a 3D printer is provided in Figure 15.

### 4.2. Stereolithography (SLA)

**Figure 16:**
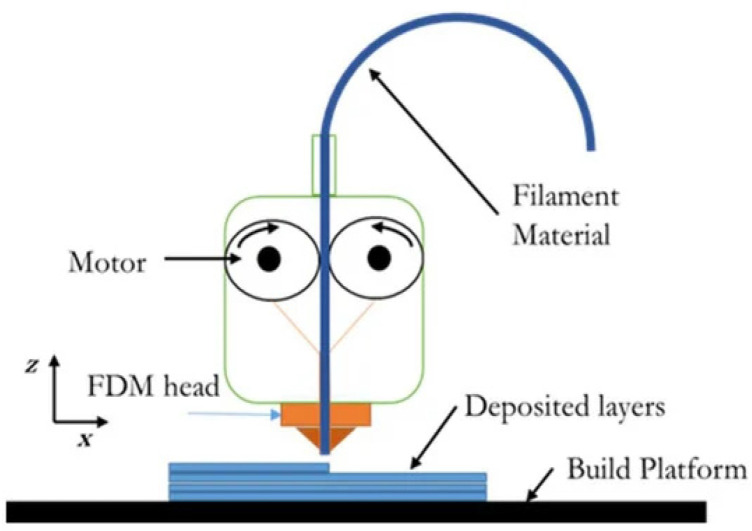
Scheme of SLA equipment. Adapted from [64].

Apart from extrusion-based techniques, other AM technologies are based on vat polymerization. Herein, the processed materials are not required to be filament-shaped. Instead, photocurable resins at the liquid state are used. Stereolithography (SLA) is a vat polymerization technique in which the photocrosslinking of each layer is achieved by indirect exposure to an ultraviolet (UV) light laser beam [63]. Figure 16 shows a representation of SLA equipment.

Briefly, the SLA apparatus comprises a vat recipient with an integrated elevator that can operate from bottom-up or top-down, according to the design of the SLA equipment [65]. It is worth mentioning that the elevator movement steps tune the layer height of the processed part. Initially, the elevator is placed where only one layer of resin is at the surface to be photopolymerized. After the equipment is fed with the photocurable resin, a UV laser beam, reflected by a movable mirror, selectively scans the first layer with the previously design layout created on CAD and slicer software [66]. The UV light acts as a photopolymerization agent, which leads to the photocrosslinking of the polymeric chains and consequent solidification and hardening of the structure. Since crosslinking is an irreversible reaction, the material cannot revert to the liquid state after solidification even when in contact with the uncured resin.

For this reason, a photocured layer can be exposed to uncured material without changing shape. The printing process continues with the dislocation of the elevator one step down or up, leading to the exposure of a new layer of uncured resin to the UV light. By the end of the printing process, the produced parts are again irradiated by UV to enhance their thermal and mechanical properties. In addition, printed parts may require post-processing for the removal of supportive structures.

Unlike FDM, the materials for SLA cannot be thermoplastic. Instead, the technique is more appropriate for thermoset polymers, pre-hydrogel mixtures, and ceramics [67,68,69]. The most attractive advantages reported using SLA are the smooth surface finishing and dimensional precision and accuracy [70].

### 4.3. Digital Light Processing (DLP)

**Figure 17:**
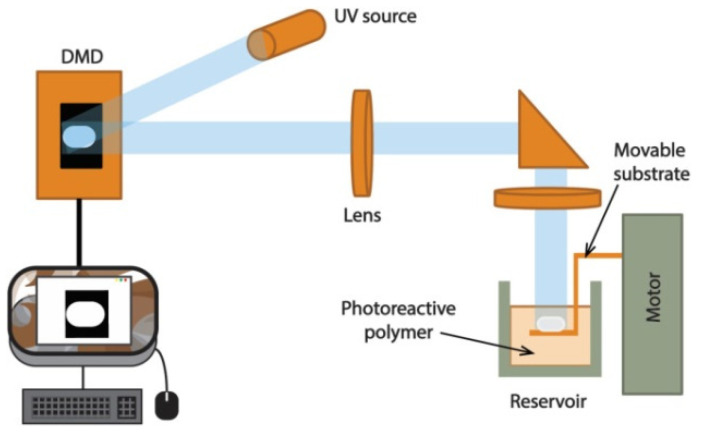
DLP equipment scheme [71]. Reprinted with permission from Elsevier.

Digital light processing (DLP) is another vat polymerization technique whose work principle is similar to SLA. However, in DLP, instead of a laser and moving mirror system, light is provided by a digital projector (Figure 17).

The projector is equipped with systems able to generate masks such as liquid crystal displays (LCD), digital micromirror device (DMD), or liquid crystal on silicon (LcoS) to transfer the exact design of each layer [72]. By using a mask system, each layer is photocured at once, which inevitably increases the processing speed. Unfortunately, only photocurable materials can be processed by DLP. Therefore, DLP is considered to be faster than SLA without jeopardizing the smooth finishing [73]. Moreover, due to the possibility of using masks with different densities of the projected design, DLP offers the possibility of producing parts with gradients of crosslinking, unlike SLA, where all the parts present the same percentage of curing.

### 4.4. EAPs Processed by Non-Conventional Technologies

#### 4.4.1. Shape Memory Polymers 

Shape memory polymers (SMPs) are materials that can change their shape or behavior when exposed to an external stimulus [74]. Such a response can be programmed based on the stimulus to pre-define the temporary shape. Most of the SMPs already processed by AM do not present electroactive properties and, therefore, it is common to incorporate responsive particles into polymeric blends, forming polymer-based electroactive composites. In this context, PLA, a thermosensitive polymer commonly used in AM due to its extrudability and shape memory properties, was blended with carbon nanotubes (CNT) to prepare electroactive composites [75]. The PLA-based composite was acquired in a filament shape and then processed by FFF. The 4D effect was optimized by varying printing parameters such as layer height, printing speed, infill pattern, and angle (0°, 45°, and 90°). It was proved that printed specimens could shape morph by applying DC voltage, and the recovery rate from temporary to original shape was influenced by the infill degree.

Following a similar approach, Zeng et al. used a dual nozzle 3D printer to print composite parts based on PLA reinforced with continuous carbon fiber [76]. The mechanical performance of the printed parts was evaluated through three-point bending tests and correlated with the printing parameters. In what concerns shape morphing, the printed parts were activated by an electric field that, based on the joule heating effect, could bend due to the increase of temperature. Herein, the metallic counterparts of the composite acted as a heating enhancer, which contributed to the faster increase of temperature in the printed part. When the glass transition temperature of PLA was surpassed, the material could bend into a temporary shape. After a shape morphing cycle, the shape recovery ratio was around 95%, proving the reversibility of the process.

**Figure 18:**
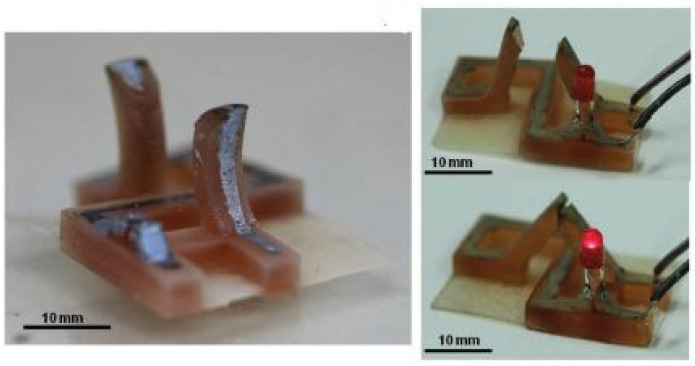
PCL/conductive ink printed parts (**left**) and printed part actuation with a temperature sensor (**right**) [77]. Reproduced with permission from Wiley-VCH.

Another reported strategy to induce electroactivity in 3D printed SMPs, consists of printing a conductive material on top of the previously SMP printed part. As proof of concept, Zarek et al. described the preparation of 4D printed parts based on PCL with a CNT-based conductive [77]. In this work, before applying the electrical stimulus, the authors programmed the shape change of the printed part according to the melting temperature of the PCL counterpart. Then, an electrical stimulus was applied, causing the heating of the structure. A temperature sensor was incorporated to signal when the temperature would surpass the melting temperature of PCL, and consequently, the material would initialize its shape morphing. When the electrical stimulus is turned off, the temperature decreases and the printed part recovers its original shape (Figure 18).

As a result of the proposed strategy, even though the shape morphing of the printed parts is based on temperature and the conformation of the polymeric network, the shape change can be triggered not only by temperature but also by applying a 50 V voltage.

**Figure 19:**
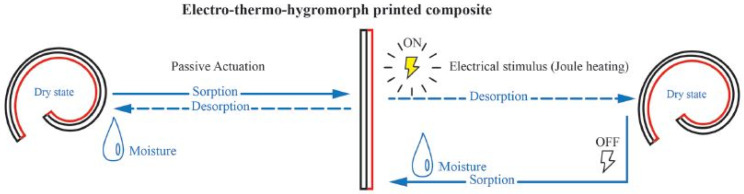
Schematic representation of actuation routes of bilayered structures where the black layer refers to PA66/carbon fibers layer (electrical response), and the red layer represents the PA66 layer (thermo and hygro response) [79]. Reproduced with permission from Wiley-VCH.

The concept of multilayers parts to achieve electro actuation was also investigated using polyamide 66 (PA66) combined with carbon fibers [78,79]. In the first approach, the FFF printed parts were composed of two layers, the base one composed of PA66, while the top was based on a mixture of PA66 with carbon fibers [78]. Regarding the design, the two layers had different geometries to enable the prediction of the temporary shape. The electro actuation was validated by the application of 12.6 W electrical power. The second work reports the fabrication of bilayered structures that, similarly to the aforementioned study, exhibit electro-thermal-hydromorphic behavior, as schematized in Figure 19.

Shape morphing can be achieved by two different mechanisms, starting from a flat bilayered printed structure. The first is related to moisture which causes the hydrophilic PA66 network to swell and is referred to as the passive actuation. On the other hand, in the second case, applying an electrical stimulus increases the temperature of the PA66/carbon fiber layer (Joule heating effect), causing the drying of the previously swelled PA66 layer. As a result of the loss of water, the PA66 layer contracts. The original shape is then recovered by turning off the electrical signal and re-exposing the printed part to a moist environment.

Currently, many shape memory polymers are already available in the market that display conductive properties. In a work conducted by Garces et al., a graphene/PLA conductive filament was printed concomitantly with a commercial shape memory polyurethane [80]. The design strategy involved creating a conductive core inside a shape memory polyurethane matrix to combine both shape memory features with electro actuation.

#### 4.4.2. Hydrogels

Hydrogels are characterized by a high capacity to uptake water and swelling, which causes their volume to expand and, consequently, their shape change. Even though the shape morphing cycles are governed by the moisture of the surrounding environment, such a response can be triggered by electrical stimuli. Such a possibility was discussed in a study where electroactive hydrogel formulations based on acrylic acid (AA) and PEG were synthesized [81].

**Figure 20:**
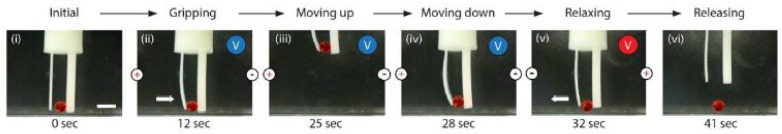
Actuation scheme of AA/PEG hydrogel under electrical stimulation [81]. Reprinted with permission from the American Chemical Society.

Then, using a vat polymerization AM technique, different parts were created and tested for 4D effect using PBS as an electrolyte. When the printed hydrogels are exposed to the electrolyte, the carboxylic groups ionize, turning the polymeric network anionic. Consequently, cations are released into the PBS solution generating an osmotic pressure gradient, which triggers the diffusion of water molecules into the polymer network. Then, by applying an electrical field (0.58 V/mm), it is possible to control the bending direction of the hydrogel (Figure 20) and, therefore, the reversible shape morphing, according to the aforementioned enrichment/depletion mechanism.

Based on the same actuation mechanism, the performance of 3D-printed chitosan was compared with chitosan and gelatin parts prepared by solvent casting [82]. The printing parameters of chitosan were extensively studied and optimized to achieve different designs with geometrical quality. As regards electrical actuation, 3D-printed chitosan proved to have a higher deflection rate compared with both chitosan and gelatin cast films while maintaining ultimate deflection values in the same range of the casted samples. Furthermore, regarding mechanical properties, 3D-printed parts delivered the highest tensile strength values and bending resistance.

This study proved that 3D printing could be a good alternative for processing natural-based polymers and increase their applicability due to the geometrical freedom and enhancement of mechanical properties. Nonetheless, the geometrical accuracy still requires improvement.

#### 4.4.3. Other Polymeric Materials

Despite shape-memory materials and hydrogels, other alternatives have been studied for the fabrication of 3D-printed EAPs. In this context, liquid crystal elastomers (LCE) might be of significant interest since they can be responsive to the electrical field and have unique elastic properties [83]. The preparation of 3D-printed LCE-based objects was described in the literature [84]. The 3D-printed objects consisted of a three-layered structure where the first layer was the commercial filament TangoBlack^TM^, the middle layer was silver ink (conductive material), and finally, the top layer was the LCE. The current application led to the shape change of the entire structure due to the contraction of the LCE polymeric network caused by the increase of temperature by the Joule heating effect. During the shape morphing cycles, no delamination defects were observed, proving that LCE-based structures may be of central interest in preparing 4D printing applications.

With the continuous dissemination of additive manufacturing, new studies have been published using available commercial products usually processed by conventional techniques [85] as photocurable materials. In a work conducted by Mu and co-workers, an acrylic-based photocurable resin was blended with multi-walled carbon nanotubes (MWCNTs) and printed using a vat polymerization technique [86]. The concentration of MWCNT was tuned to enhance the conductivity of the blends without jeopardizing the rheology properties, being the optimal loading around 0.3 wt%. Incorporating the MWCNTs contributed to the slight increase of the modulus and ultimate tensile strength and, therefore, the stretchability of the printed parts decreased. Due to the joule heating effect, the printed parts could reversibly shape morph when exposed to an electrical stimulus.

The field of electronics is constantly evolving in a direction where the size of the devices should be as small as possible. AM might be especially interesting in this regard as it enables the fabrication of reduced intricate shapes with high geometric accuracy. In an attempt to test the limits of extrusion-based AM techniques, a team of researchers built a custom 3D printer that allowed the fabrication of PPy electroactive parts within the micrometer range [87].

## 5. Conclusions and Future Perspectives

The following main mechanisms can describe the electromechanical displacements (bending motion) of EAP actuators: Coulomb, electroosmosis, electrochemical, enrichment/depletion, converse piezoelectric effect, EDL formation, and cation-/anion-driven mechanism. In EAP electromechanical sensors, the main mechanisms responsible for their behavior are the direct piezoelectric effect and uneven ion distribution. Regarding EAP-based drug-delivery systems, its response is described by the forced convention mechanism. The type of mechanism that adequately describes the electromechanical response of an EAP is dependent on its physicochemical nature/composition but not restricted by it.

Most of the current EAPs developed by conventional processing techniques rely on well-explored electro-responsive polymers, including PPy, PEDOT:PSS, PANI, PVDF, and Nafion^TM^. However, a significant effort has been made to expand this array of materials by utilizing PILs to develop unique ionic EAPs with distinct properties. Moreover, natural polymer-based EAPs with excellent performance were also obtained by conjugating polysaccharides with the aforementioned conducting polymers, CNTs, and additives such as free ions. Due to the diverse array of processing variables, namely size/shape, porosity, conductivity, and mechanical properties, it is impossible to establish a fair comparison in terms of the electro-assisted performance among these materials.

The processing of EAPs by AM is still in an early stage of development. For this reason, the focus of research in this field is studying the processability of materials and their effect on electroactive properties. Researchers combine non-conductive materials with metallic particles or create multilayered structures to induce shape morphing after exposure to an electrical stimulus to overcome the fact that AM cannot process most conventionally processed EAPs due to rheological problems. In addition, the control over the shape morphing still represents a challenge regarding the reproducibility of the bending angles and recovery of the original shapes. Nonetheless, this field of research is of central interest, especially for soft robotics, since AM processing enables the production of small parts with intricate shapes and details.

Despite the significant amount of work published in the literature for EAPs, there is still plenty of opportunities/challenges to attain/overcome in terms of sustainable processability, fast and accurate electromechanical response over repetitive cycles and in different environments, electrochemical resistance, mechanical properties, and in some specific cases, biocompatibility, and non-toxicity. Following the increased number of publications on 3D and 4D printing, it is expected that the study of new EAPs processed by AM will be one of the hot topics in the future.

## Data Availability

No new data was produced in this manuscript. Not applicable.
